# Effects of Black Soldier Fly Larvae Hydrolysate on Culture of Primary Myogenic and Adipogenic Cells Isolated from Broilers for Cultured Meat Development

**DOI:** 10.3390/foods14040678

**Published:** 2025-02-17

**Authors:** Sang-Hun Park, Se-Hyuk Oh, Gyu-Tae Park, So-Young Jang, Young-Ho Lim, Sung-Kyun Oh, Tae-Hyung Lee, Sol-Hee Lee, Jong-Hyuk Kim, Jung-Seok Choi

**Affiliations:** Department of Animal Science, Chungbuk National University, Cheongju 28644, Republic of Korea; pksaho@chungbuk.ac.kr (S.-H.P.); osehyuk12@naver.com (S.-H.O.); qkrrbxo114@chungbuk.ac.kr (G.-T.P.); soyoung01021@chungbuk.ac.kr (S.-Y.J.); 0ho0704@chungbuk.ac.kr (Y.-H.L.); sk0630@chungbuk.ac.kr (S.-K.O.); jijone@dodram.co.kr (T.-H.L.); leesh73@chungbuk.ac.kr (S.-H.L.); jonghyuk@chungbuk.ac.kr (J.-H.K.)

**Keywords:** black soldier fly larvae hydrolysate, broiler primary cells, myogenic cells, adipogenic cells, stromal vascular fractions, cultured meat

## Abstract

Sustainable food resources, including cell-cultured meat and edible insect proteins, are emerging as key solutions to meet future protein demands. This study evaluated the effects of black soldier fly larvae hydrolysate (BLH) on primary cells isolated from broiler leg and breast muscle tissues, as well as abdominal fat tissues. Primary cells isolated from each tissue were characterized for their myogenic and adipogenic (stromal vascular fraction, SVF) properties. Cells were cultured in a basal medium with five percent FBS supplemented with BLH at concentrations ranging from 25 to 300 µg/mL. Leg and breast muscle cells showed significantly enhanced proliferation, as indicated by MTS assay results and cell counts, in the BLH100 group compared to the FBS5 and control groups (*p* < 0.05). Furthermore, the expression of myogenic markers, including PAX7, NCAM1, MYF5, and MYOD1, was upregulated in leg muscle cells treated with BLH (*p* < 0.05). For SVFs, BLH50 promoted cell proliferation; however, differentiation decreased as BLH concentration increased. These findings suggest that BLH can enhance the proliferation of primary broiler cells, highlighting its potential applicability in the edible insect and cultured meat industries.

## 1. Introduction

The projected increase in the global population to over 9 billion by 2050 intensifies the demand for sustainable meat production and protein sources [1]. Consequently, efficient and sustainable alternatives are needed to solve the problem of a partially insufficient protein supply [2]. While cell-cultured meat and edible insect proteins have been separately recognized as promising alternatives [3], there is a critical gap in exploring their potential integration. This study focuses on black soldier fly larvae hydrolysate (BLH) as a resource for culturing primary broiler cells, addressing food sustainability, and innovative protein production.

The development of cell-cultured meat technology is gaining attention as a solution to future food and meat production sustainability issues [4]. This futuristic food production technology involves culturing livestock somatic cells in vitro to replicate and mimic traditional meat. Cell-cultured meat has the potential to produce meat without slaughtering animals, addressing animal ethics concerns and supplementing scarce protein resources, thereby maintaining food sustainability [5]. Moreover, advances in this technology could alleviate issues such as antibiotic resistance in livestock and foodborne diseases, which are obstacles to food sustainability [6,7]. This field has the potential to create economic, social, and religious value by supporting food aid for underdeveloped and developing countries while also having the potential to reach the vegan market. Further research in this area is well justified [8,9,10]. Therefore, active research is being conducted from an economic production perspective to advance the development and industrialization of cell-cultured meat, and related research should be undertaken widely across various fields [11,12]. However, current production systems face significant challenges, including high costs, a lack of alternative growth media, and the absence of established continuous production systems. Addressing these limitations is essential for advancing the commercialization of cultured meat [13].

Edible insects are considered desirable food sources due to their nutritional profiles and low environmental footprints. Industrial-scale insect farming requires less water and land than pasture-based insect farming. Edible insects, including black soldier fly larvae, can be raised on various organic wastes such as food scraps, straw, and animal intestines. This enables resource recycling by feeding them to larger livestock [14,15]. The edible insects also have high protein contents that are similar to animal proteins. They contain all the essential amino acids required by humans [16]. Black soldier fly larvae are a rich source of proteins, containing 37–63% dry matter. Due to their nutritional value, black soldier fly larvae are being utilized for medicine, broiler feed, and fish feed [17,18,19,20,21]. In the European Union, the Novel Food Regulation (Regulation (EU) 2015/2283) governs the market introduction of new foods, including insects. This regulation, effective from 1 January 2018, mandates the safety assessment of novel ingredients not previously used as food. Black soldier fly larvae are classified as Novel Food under this regulation and require safety evaluation and approval from the European Food Safety Authority (EFSA) before being marketed [22]. In South Korea, black soldier fly larvae were officially defined as livestock that can contribute to farmers’ income generation in September 2023 [23]. Despite their potential as a valuable food resource, consumer acceptance faces significant barriers due to aversion or anxiety. Therefore, processed black soldier fly larvae forms are more likely to be accepted by consumers than their natural form [24]. Additionally, there is a novel method where they can be utilized in animal cell cultures for food production. Amino acids play an essential role in cell growth, survival, protein synthesis, and metabolic regulation. Black soldier fly larvae are rich in essential and non-essential amino acids, making them a promising resource for producing cultured meat and edible proteins based on cell cultures [16,25,26]. The use of BLH is expected to offer additional benefits such as cytoprotective, antioxidant, and anti-adipogenesis activities, [27,28,29]. Additionally, since the use of animal-derived resources is more effective than other resources in enhancing cell production, BLH could be a key candidate for the growth of primary cells [26,30]. This method addresses consumer concerns about consuming black soldier fly larvae while leveraging resource recycling benefits. This protein production method can contribute to developing both the edible insect and cultured meat industries. However, research studies that use edible insects to produce cultured meat are currently scarce [26,31,32].

BLH has been reported to enhance the proliferation of primary myogenic cells across various livestock species while exhibiting anti-adipogenesis activities [26,28,33]. Therefore, it can be hypothesized that BLH acts as an additive that promotes proliferation in primary myogenic cells while reducing adipogenesis in primary adipogenic cells. Thus, this study investigates the potential of BLH in supporting the growth of primary broiler cells for cultured meat production. By leveraging its rich amino acid content and bioactive properties, BLH offers a sustainable alternative to conventional growth media, addressing key barriers in cultured meat production. The findings are expected to advance the industrial scalability of cultured meat while supporting the edible insect industry, bridging the gap between these two promising fields and contributing to global food sustainability.

## 2. Materials and Methods

### 2.1. Preparation of Black Soldier Fly Larvae Hydrolysate (BLH)

The hydrolysate was prepared using commercially available defatted black soldier fly larvae powder with a particle size of 200 mesh (Entomo, Cheongju-si, Republic of Korea), which was defatted using a cold-pressing method. The defatted black soldier fly larvae powder had a crude protein content of over 55% and a crude fat content of less than 10%. First, black soldier fly larvae powder was added to pH 8 phosphate-buffered saline (PBS) (Ricca Chemical Company, Arlington, TX, USA, Cat #RCR5819600-500A) containing dissolved trypsin powder (Gibco, Waltham, MA, USA, Cat #27250018). The enzyme to black soldier fly larvae powder ratio was 1:50 (*w*/*v*). The mixture was hydrolyzed by incubating it in a water bath at 50 °C for 4 h. The hydrolysis reaction was terminated by heating the mixture to 90 °C for 10 min. Following the termination, the hydrolysate mixture was centrifuged at 4000× *g* for 20 min to separate the supernatant. The obtained supernatant was frozen and then freeze-dried to produce BLH in powder form [34].

### 2.2. Isolation of Primary Cells (Muscle Cells, Stromal Vascular Fractions (SVF)) from Chicken Tissues (Breast, Leg, and Abdominal Fat)

**Figure 1:**
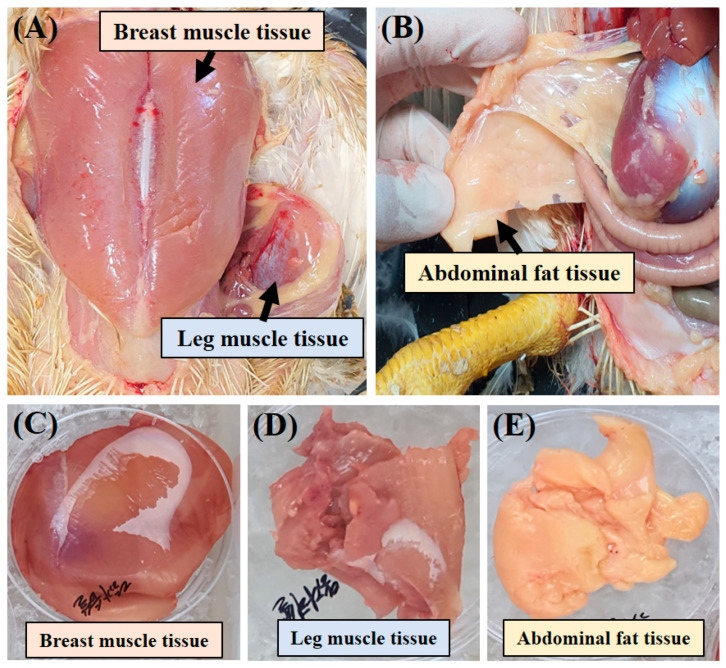
Tissue collection and locations in a 5-week-old broiler. (**A**) Locations of breast muscle tissues and leg muscle tissues in the chicken, (**B**) location of abdominal fat, (**C**) collected breast muscle tissues, (**D**) leg muscle tissues, and (**E**) abdominal fat tissues from the chicken.

**Figure 2:**
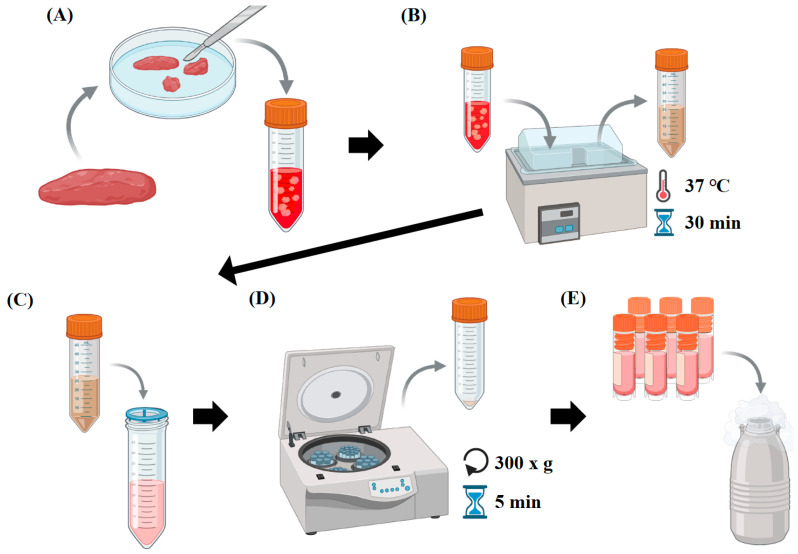
The process of primary cell isolation and cryopreservation from chicken tissues. (**A**) Physically remove other tissues (e.g., connective tissue, blood) from the chicken tissues and mince them to isolate cells; (**B**) begin proteolytic action on samples with collagenase at 37 °C for 30 min; (**C**) filter the samples through 100 µm and 40 µm strainers after ACK lysis buffer treatment; (**D**) centrifuge at 300× *g* for 5 min; (**E**) cryopreserve the isolated chicken primary cells in liquid nitrogen.

Breast muscle tissue (approximately 100 g), leg muscle tissue (approximately 50 g), and abdominal fat (approximately 30 g) were collected from a 5-week-old Ross 308 broilers post-slaughter (Figure 1). Collected tissues were enzymatically digested with collagenase type II mix (600 units/mL DMEM) (Worthington, Lakewood, NJ, USA, Cat # LS004176) at 37 °C for 30 min and repeated centrifugation at 70× *g* and 800× *g* to remove connective tissues. These cell pellets were treated with ACK (Ammonium Chloride–Potassium) lysis buffer to remove red blood cells and then filtered through 100 µm and 40 µm filters. These isolated cells were stored in liquid nitrogen (Figure 2) [35].

### 2.3. Pre-Plating of Primary Cells Isolated from Chicken Breast and Leg Muscle Tissues

**Figure 3:**
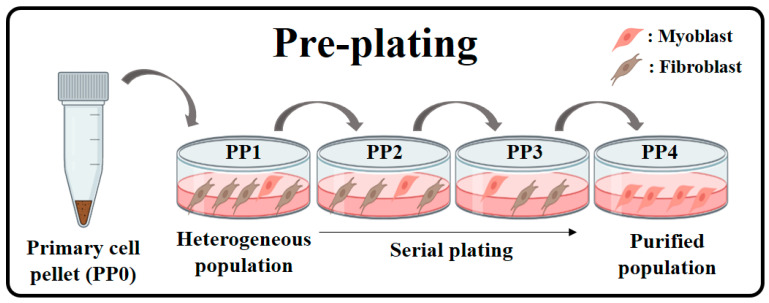
Purification of chicken leg- and breast-derived myogenic cells through pre-plating.

Primary cells isolated from chicken breast muscle tissue and leg muscle tissue were subjected to the pre-plating (PP) technique to purify satellite cells. Cells (PP0) derived from chicken muscles were seeded into flasks pre-coated with collagen (collagen solutions, Cat # FS22005). After a 30 min incubation (PP1), the supernatant was transferred to a new collagen-coated flask. This process was repeated at 60 min (PP2), 90 min (PP3), and 120 min (PP4) consecutively. Chicken cells which adhered to the flasks were designated as the PP1 through PP4 groups (Figure 3). These cells were cultured in a sample of Ham’s F-10 nutrient mix (Gibco, Cat # 11550043) supplemented with 10% FBS (fetal bovine serum) and 1% PSA (penicillin–streptomycin–amphotericin B solution, Lonza, Basel, Switzerland, Cat # 17745E). Cultures were maintained in a humidified incubator at 37 °C with 5% CO_2_.

### 2.4. Culturing and Imaging of Chicken Leg- and Breast-Derived Myogenic Cells and SVFs

Purified chicken breast myogenic cells and leg myogenic cells were seeded into collagen-coated flasks. These muscle cells were cultured in a sample of Ham’s F-10 nutrient mix (Gibco, Cat # 11550043) supplemented with 5% or 10% FBS and 1% PSA (penicillin–streptomycin–amphotericin B solution, Lonza, Cat # 17745E), referred to as FBS5 and FBS10, respectively. BLH was supplemented to the FBS5 group at concentrations of 25 µg/mL (BLH25), 50 µg/mL (BLH50), 100 µg/mL (BLH100), 200 µg/mL (BLH200), or 300 µg/mL (BLH300). SVFs isolated from chicken abdominal fat tissues were also seeded into collagen-coated flasks. For the proliferation culture, SVFs were cultured in a sample of Dulbecco’s modified eagle’s medium (DMEM) supplemented with 5% FBS and 1% PSA, referred to as the SFBS5. For the differentiation culture, once SVF cells reached approximately 70% confluence, the medium was replaced with a differentiation medium consisting of DMEM supplemented with 5% FBS, 1% PSA, 10 µg/mL insulin, 0.5 mM 3-isobutyl-1-methylxanthine (IBMX), 1 µM dexamethasone, and 5 µM rosiglitazone. Cells were then cultured in this medium for 3 days. After 3 days, the medium was replaced with a differentiation medium excluding IBMX and dexamethasone. Concentrations of BLH added to the SFBS5 were the same for both the proliferation and differentiation cultures of SVFs. BLH was supplemented to the SFBS5 group at concentrations of 25 µg/mL (SBLH25), 50 µg/mL (SBLH50), 100 µg/mL (SBLH100), 200 µg/mL (SBLH200), or 300 µg/mL (SBLH300). All chicken cells were cultured in a humidified incubator at 37 °C with 5% CO_2_. These cultured cells were observed daily using an optical microscope (EVOS-5000, Thermo Fisher, Waltham, MA, USA), and brightfield images were then captured.

### 2.5. Cell Counting and Viability Measurement

Cells cultured in collagen-coated flasks, including chicken breast muscle cells, leg muscle cells, and SVF cells, were detached using trypsin-EDTA. Trypsin activity was neutralized with 2% FBS in PBS solution. Live cell count and viability were measured using an automated cell counter (Countess Cell FL Automated Cell Counter, Invitrogen, Waltham, MA, USA).

### 2.6. Cell Proliferation Assay (MTS)

Cell proliferation was assessed using a CellTiter 96^®^ AQueous One Solution Cell Proliferation Assay (Promega, Madison, WI, USA). After culturing cells on collagen-coated microplates, 20 µL of the MTS colorimetric reagent was added to each well containing 100 µL of the medium. Plates were incubated at 37 °C for 2 h in a 5% CO_2_ atmosphere. Absorbance was measured at 490 nm.

### 2.7. Oil Red O Staining and Quantification

To perform Oil Red O staining, differentiated SVFs were first fixed with 2% paraformaldehyde for 1 h and subsequently washed with PBS. After fixation, 70% isopropanol was added and incubated for 5 min. The staining solution was prepared by mixing Oil Red O solution with distilled water at a 3:2 ratio and filtering it through a 0.45 μm syringe filter (6784-2504, Whatman, Maidstone, UK). The cells were then incubated at 37 °C for 20 min in the prepared Oil Red O working solution. Following incubation, the staining solution was removed and the cells were washed thoroughly with distilled water five times before microscopic observation.

For quantification, SVFs were gently rinsed three times with 70% isopropanol. To extract the Oil Red O dye retained in the cells, 100% isopropanol was added and the plate was shaken gently for 5 min. The absorbance of the extracted dye was measured at 492 nm using a spectrophotometer.

### 2.8. Nile Red Staining

For Nile Red staining, the culture medium was removed from differentiated SVFs and the cells were washed twice with PBS. The cells were then fixed in 2% paraformaldehyde for 1 h. After fixation, paraformaldehyde was removed, and the SVFs were washed twice with distilled water. Nile Red dye (GC15539, GLPBIO, Montclair, CA, USA) was diluted in PBS to a final concentration of 1 μg/mL to prepare the working solution. The cells were then treated with this solution and incubated at room temperature for 30 min.

### 2.9. RT-qPCR

To analyze gene expression, cultured cells were harvested and total RNA was extracted using a Total RNA Extraction Kit (iNtRON Biotechnology, Seongnam-si, Republic of Korea, Cat # 17221, Korea), following the manufacturer’s protocol. Complementary DNA (cDNA) synthesis was carried out using the High-Capacity cDNA Reverse Transcription Kit (Thermo Fisher, Cat # 4368814). Quantitative real-time PCR (RT-qPCR) was performed with Fast qPCR 2 × SYBR Green Master Mix (Cat # EBT-1821). The amplification conditions included an initial step at 50 °C for 2 min and 95 °C for 10 min, followed by 40 cycles of 95 °C for 15 s and annealing at 52–55 °C for 1 min. The genes analyzed were *PDGFRA*, *ITGB1*, *MYOD1*, *PAX7*, *NCAM1*, *MYF5*, *CEBPB*, and *ACTB*. The primer sequences used for amplification are listed in Table 1. Gene expression levels were calculated using the 2−ΔΔCT method [36].

### 2.10. Statistical Analysis

Each experiment was performed in triplicate. For myogenic and adipogenic cell experiments, three biological replicates (n = 3, independent cell isolations) were used. The results are presented as mean ± standard deviation (SD). Statistical analyses were performed using a one-way analysis of variance (ANOVA) with the General Linear Model (GLM) procedure in SAS software (Statistical Analysis System, version 9.4, 2002, Cary, NC, USA). A Duncan’s multiple range test was applied to determine significant differences among the treatment group mean, with statistical significance set at (*p* < 0.05).

### 2.11. Ethics Approvals

The Institutional Animal Care and Use Committee (IACUC) of Chungbuk National University approved the animal study protocol (CBNUA-2107-23-01).

## 3. Results and Discussion

### 3.1. Purification of Myogenic Cells Through Pre-Plating

**Figure 4:**
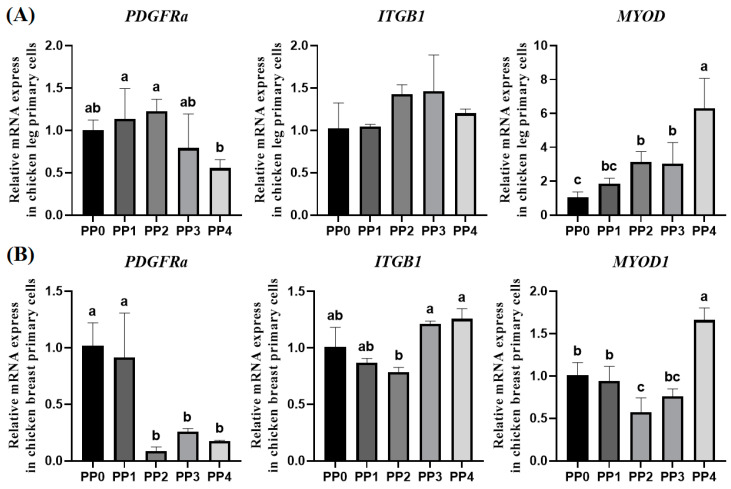
RT-qPCR evaluation of pre-plating enriched chicken primary leg and breast myogenic cells. (**A**) The relative mRNA levels of *PDGFRA*, *ITGB1*, and *MYOD1* in chicken leg myogenic cells following pre-plating and (**B**) relative mRNA levels of *PDGFRA*, *ITGB1*, and *MYOD1* in chicken breast myogenic cells following pre-plating. *ACTB* was used as the reference gene. Data are presented as mean ± SD from three biological replicates (n = 3). Different letters (a–c) indicate significant differences (*p* < 0.05). PP: Pre-plating.

Primary cells isolated from chicken leg and breast muscle tissues were purified using the pre-plating method, as illustrated in Figure 3. This method exploits the differential adhesion times of fibroblasts and satellite cells or myoblasts. Generally, satellite cells or myoblasts take longer to adhere than fibroblasts. Fibroblasts bind more strongly to key ECM components such as collagen, laminin, and fibronectin than satellite cells. In contrast, satellite cells and myoblasts rely more on cell–cell adhesion than ECM binding. As satellite cells and myoblasts differentiate, the expression of cell–cell adhesion molecules and activation of ECM receptors such as integrins enhance their binding to the ECM [37]. Following muscle cell seeding, the supernatant was transferred to a new flask at specific intervals to define treatment groups. The level of muscle cell purification was assessed using markers specific to fibroblasts and myogenic cells. Both chicken leg- and breast-derived myogenic cells showed a decreasing trend in the relative amount of *PDGFRA* mRNA, a fibroblast-specific marker, as pre-plating progressed (*p* < 0.05). *PDGFRA* mRNA was significantly reduced in the PP4 group in cells derived from leg muscle and in the PP2 group in cells derived from breast muscle (*p* < 0.05). The relative amount of *ITGB1* mRNA was significantly higher in the PP3 and PP4 groups of cells derived from leg muscle, while there were no significant differences among treatment groups for cells derived from breast muscle. Various studies have reported high ITGB1 expression in satellite cells, and ITGB1 is recognized as a key marker for satellite cell activation and muscle stem cells in muscle regeneration research [38]. Additionally, in cell purification using FACS, the ITGB1 marker has been employed to distinguish both satellite cells and fibro-adipogenic progenitors [39]. The relative amount of the myoblast-specific marker *MYOD1* mRNA tended to increase with pre-plating progression (*p* < 0.05). Several pre-plating studies have compared the purity of satellite cells and fibroblasts by analyzing the MYOD and PDGFRα markers [40]. When considering only the purification of fibroblasts, cells derived from leg muscle can be selected from the PP4 group, while cells derived from breast muscle have the option of being selected from the PP2 group. In this study, considering levels of fibroblasts and myoblasts, chicken leg and breast myogenic cells from the PP4 group were used for subsequent experiments (Figure 4A,B). Additionally, the *ACTB* gene used in this experiment is widely recognized as a stable reference gene in chicken cells [41,42].

### 3.2. Proliferation of Pre-Plated Chicken Leg- and Breast-Derived Primary Myogenic Cells Induced by BLH

**Figure 5:**
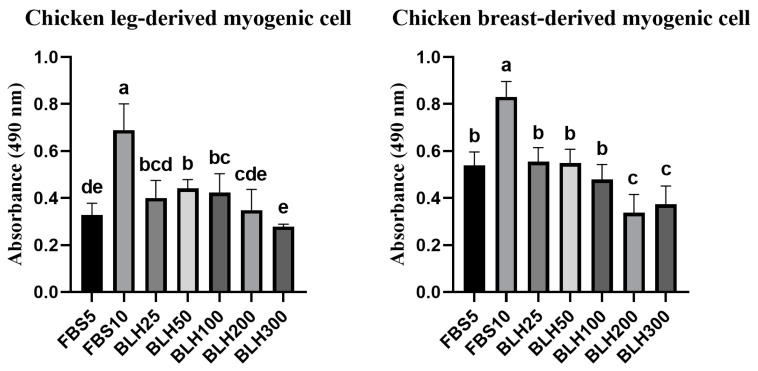
MTS assay of chicken leg- and breast-derived myogenic cells cultured for 3 days with the addition of BLH. Data are presented as mean ± SD from three biological replicates (n = 3). Different letters (a–e) indicate significant differences (*p* < 0.05). FBS5: Ham’s F10 + 5% FBS; FBS10: Ham’s F10 + 10% FBS; BLH25: FBS5 + 25 µg/mL BLH; BLH50: FBS5 + 50 µg/mL BLH; BLH100: FBS5 + 100 µg/mL BLH; BLH200: FBS5 + 200 µg/mL BLH; BLH300: FBS5 + 300 µg/mL BLH.

To measure the effect of BLH on the proliferation of chicken leg- and breast-derived primary myogenic cells, both cell types were cultured for 3 days and subjected to an MTS assay (Figure 5). For leg-derived myogenic cells, all treated groups had lower absorbance values than the FBS10 group. BLH50 and BLH100 groups showed significantly higher cell proliferation than the FBS5 group (*p* < 0.05). Increasing concentrations of the BLH led to a decrease in cell proliferation. For breast myogenic cells, all treated groups had lower absorbance values than the FBS10 group (*p* < 0.05). These values of all treated groups were equal to or lower than those of the FBS5 group. This study revealed that BLH at concentrations of 25–100 µg/mL was not toxic to chicken myogenic cells; instead, it may enhance their proliferation at low concentrations. However, in breast-derived myogenic cells, treatment groups such as BLH200 and BLH300 showed lower proliferation compared to FBS5.

**Figure 6:**
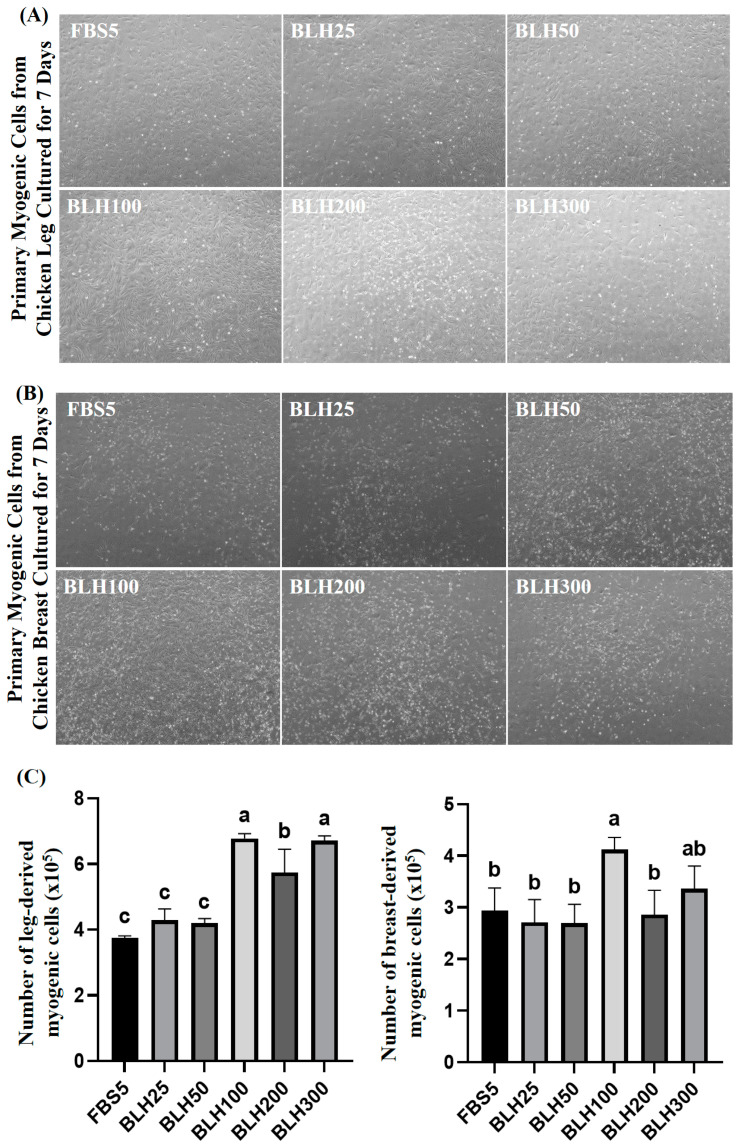
Proliferation assay of chicken leg- and breast-derived myogenic cells cultured for 7 days with the addition of BLH. Microscopic images of chicken leg- (**A**) and breast-derived (**B**) myogenic cells (magnification: ×40). (**C**) Cell count measurements of chicken leg- and breast-derived myogenic cells. Data are presented as mean ± SD from three biological replicates (n = 3). Different letters (a–c) indicate significant differences (*p* < 0.05). FBS5: Ham’s F10 + 5% FBS; BLH25: FBS5 + 25 µg/mL BLH; BLH50: FBS5 + 50 µg/mL BLH; BLH100: FBS5 + 100 µg/mL BLH; BLH200: FBS5 + 200 µg/mL BLH; BLH300: FBS5 + 300 µg/mL BLH.

Figure 6A,B displays images of chicken leg- and breast-derived primary myogenic cells cultured for 7 days with varying concentrations of BLH. Figure 6C shows the number of chicken leg- and breast-derived myogenic cells cultured for 7 days. BLH100, BLH200, and BLH300 groups had significantly higher numbers of leg-derived myogenic cells than the FBS5 group (*p* < 0.05). Similarly, the number of breast-derived myogenic cells in the BLH100 group was significantly higher than that in the FBS5 group (*p* < 0.05). BLH100 enhanced the cell counts of both leg- and breast-derived myogenic cells. Figure 5 and Figure 6C suggest that, while the addition of BLH did not affect cell proliferation during the 3-day culture of chicken myogenic cells, a noticeable effect was observed at specific concentrations after at least 7 days of culture. This indicates that BLH exhibits significant effects on cell proliferation at 7 days of culture.

**Figure 7:**
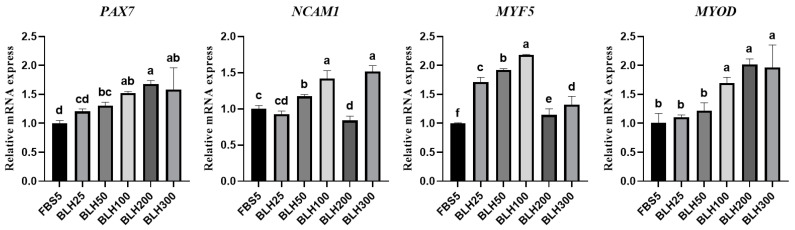
Relative mRNA (*PAX7*, *NCAM1*, *MYF5*, *MYOD1*) expression levels in a proliferation analysis of chicken leg-derived myogenic cells cultured for 7 days with the addition of BLH at different concentrations. *ACTB* was used as the reference gene. Data are presented as mean ± SD from three biological replicates (n = 3). Different letters (a–f) indicate significant differences (*p* < 0.05). FBS5: Ham’s F10 + 5% FBS; BLH25: FBS5 + 25 µg/mL BLH; BLH50: FBS5 + 50 µg/mL BLH; BLH100: FBS5 + 100 µg/mL BLH; BLH200: FBS5 + 200 µg/mL BLH; BLH300: FBS5 + 300 µg/mL BLH.

Expression levels of genes related to muscle cell-specific markers and myogenic cell proliferation were analyzed to measure the effect of BLH on the proliferation of chicken leg-derived myogenic cells (Figure 7). The relative mRNA levels of the muscle-specific gene *PAX7* were higher in the BLH50, BLH100, BLH200, and BLH300 groups than in the FBS5 group (*p* < 0.05). The relative mRNA levels of the satellite cell marker *NCAM1* were higher in the BLH100 and BLH300 groups than in the FBS5 group (*p* < 0.05). The relative mRNA levels of myogenic regulatory genes *MYF5* and *MYOD1* in all treatment groups were equal to or higher than those in the FBS5 group, with MYF5 and MYOD1 mRNA showing the highest levels in the BLH100 group. The *MYOD1* gene was highly expressed even in the BLH200 and BLH300 treatment groups. These findings suggest that BLH can upregulate genes related to cell proliferation, with BLH100 significantly upregulating *PAX7*, *NCAM1*, *MYF5*, and *MYOD1* mRNA levels. In the case of chicken leg-derived myogenic cells, the increased proliferation observed under the influence of high concentrations of BLH, such as in the BLH100, 200, and 300 treatment groups during the 7-day culture, corresponds to Figure 6C.

According to previous studies, applying BLH to cells at high concentrations for a short period reduced cell growth, whereas appropriate concentrations exhibited cytoprotective and antioxidant effects [26,29]. Additionally, studies have reported that a higher degree of hydrolysis does not necessarily lead to increased bioavailability [26,43]. The previous literature aligns with this study, suggesting that the antioxidant effects of BLH may influence the upregulation of myogenic regulatory factors [26,44,45]. In cell culture, glutamine metabolism typically leads to ammonia production, which is a major factor inhibiting cell survival and growth [46]. BLH is known to contain a high concentration of glutamic acid [26,34], which can serve as an alternative metabolic substrate to glutamine. Studies have reported that utilizing TCA cycle intermediates such as α-ketoglutarate, glutamic acid, and glutamate can reduce ammonia accumulation in the culture medium [47,48]. Additionally, glutamic acid can be directly utilized in the TCA cycle, supporting cell growth and energy supply. In long-term cultures over seven days, the glutamic acid in BLH may contribute to reducing ammonia accumulation in the medium while serving as an energy source. This explains why the BLH-treated groups with high concentrations (BLH100, BLH200, and BLH300) exhibited greater proliferation effects at seven days of culture compared to three days (Figure 5 and Figure 6C). Furthermore, during the seven-day culture period, the continuous energy supply from BLH-derived glutamic acid likely contributed to maintaining or even increasing the number of myogenic cells in BLH-treated groups compared to the FBS5 group (Figure 6C). Additionally, the amino acid composition of BLH may have encouraged cells to utilize amino acids as an energy source instead of glucose, potentially reducing lactate accumulation in the medium. This could help mitigate pH fluctuations in the culture medium and extend its lifespan [49]. Leucine and Arginine are known to upregulate mTOR signaling, which contributes to protein synthesis in muscle cells [50]. Black soldier fly larvae are known to contain these amino acids in high concentrations, and exploring the upregulation of muscle cell protein synthesis based on their amino acid composition could present a novel approach [51]. Low BLH concentrations (BHL25, BLH50) appear to enhance the proliferation of chicken myogenic cells during short-term culture, whereas high BLH concentrations (BLH100, BLH200, BLH300) seem to promote proliferation during long-term culture (Figure 5, Figure 6C and Figure 7). The current study emphasizes the need to optimize the hydrolysate concentration to avoid inhibitory effects carefully. These findings suggest that the hydrolysate has the potential to serve as a beneficial supplement for muscle cell proliferation.

### 3.3. Confirmation of Differentiation from SVF Cells to Adipocytes and the Proliferation and Differentiation of Chicken SVFs Induced by BLH

**Figure 8:**
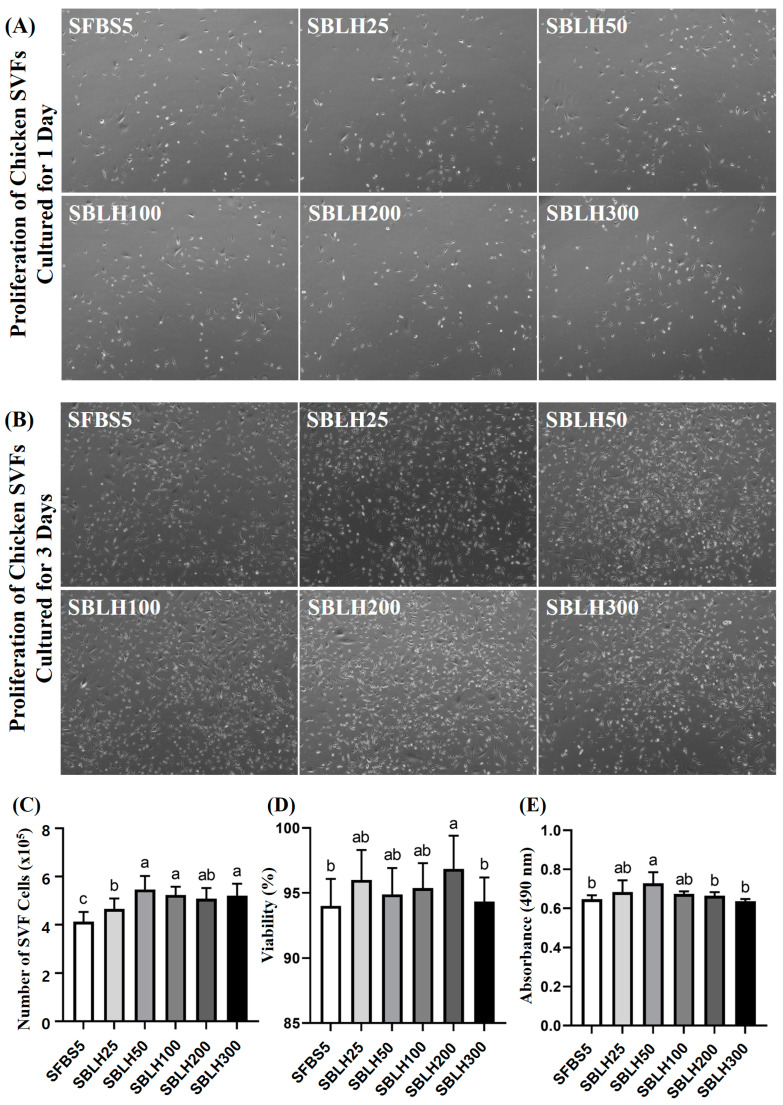
A proliferation analysis of chicken stromal vascular fractions. Microscopic images of chicken stromal vascular fractions cultured for 1 day (**A**) and 3 days (**B**) with BLH at different concentrations (magnification: ×40). The number of cells (**C**), cell viability (**D**), and the MTS (490 nm) assay (**E**) of stromal vascular fractions (SVF) with the addition of BLH. Data are presented as mean ± SD from three biological replicates (n = 3). Differ letters (a–c) indicate significant differences (*p* < 0.05). SFBS5: DMEM + 5% FBS; SBLH25: SFBS5 + 25 µg/mL BLH; SBLH50: SFBS5 + 50 µg/mL BLH; SBLH100: SFBS5 + 100 µg/mL BLH; SBLH200: SFBS5 + 200 µg/mL BLH; SBLH300: SFBS5 + 300 µg/mL BLH.

Figure 8A,B illustrates images of SVFs cultured for 1, and 3 days, respectively, with varying concentrations of BLH. Figure 8C–E presents the results of analyzing the effects of BLH on SVF proliferation, including cell count, viability, and MTS assay results. Cell count represents the number of live SVF cells after 3 days of culture, while viability indicates the proportion of live cells relative to the total cell population in each treatment group. The MTS assay is a representative proliferation assay. All BLH-treated groups had significantly higher cell counts of SVFs than the SFBS5 (*p* < 0.05). SBLH200 groups had significantly higher viability (%) of SVFs than SFBS5 (*p* < 0.05). The MTS value in the BLH50 group was significantly higher than the SFBS5 group (*p* < 0.05). It was determined that BLH concentrations of up to 300 µg/mL were not toxic to chicken SVFs. In addition, BLH at 50 µg/mL enhanced the proliferation of chicken SVFs. Similarly to chicken muscle cells, SVFs also demonstrate increased cell viability at the optimal concentration of BLH. To determine the effect of BLH on the differentiation capacity of SVF cells, confluent SVFs were supplemented with various concentrations of BLH and cultured for differentiation.

**Figure 9:**
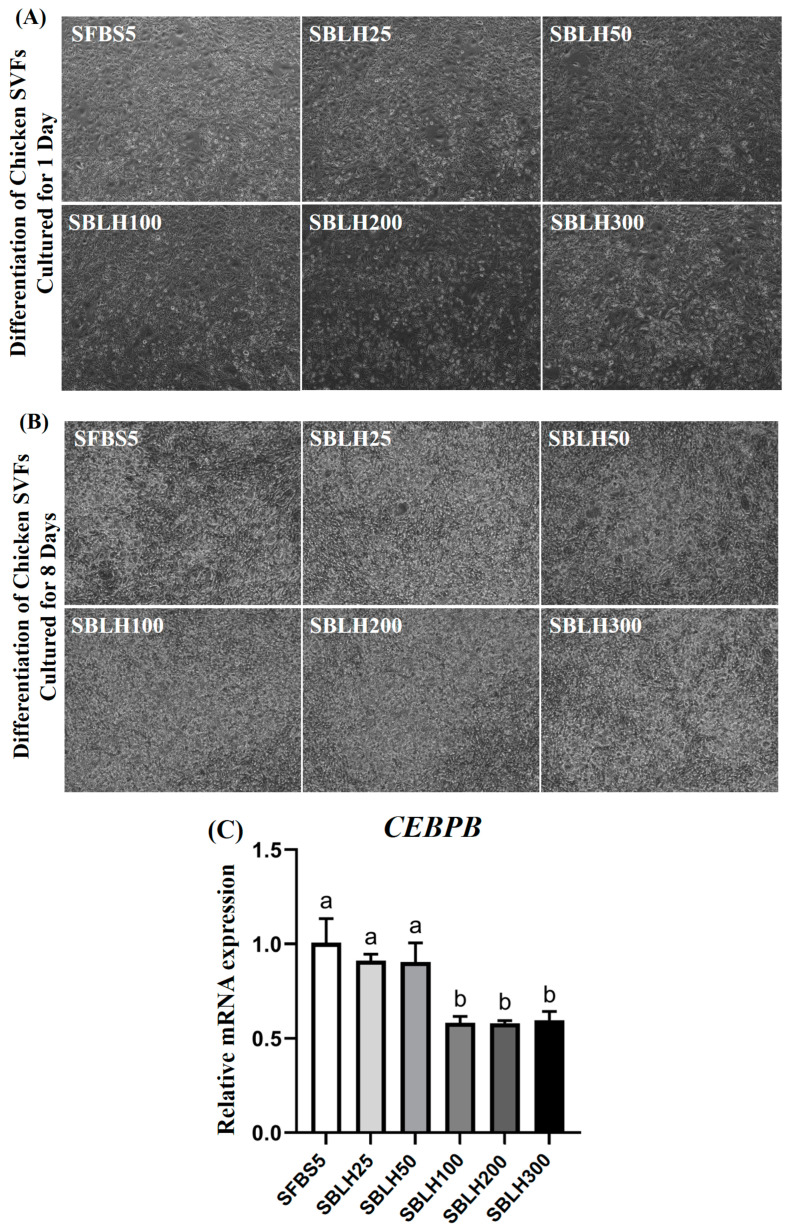
Differentiation analysis of chicken stromal vascular fractions. Microscopic images of chicken stromal vascular fractions differentiation cultured for 1 day (**A**) and 8 days (**B**) with the addition of BLH (magnification: ×40). (**C**) Relative mRNA (*CEBPB*) levels of chicken stromal vascular fractions (SVF) under differentiation conditions for 8 days with BLH treatment. Data are presented as mean ± SD from three biological replicates (n = 3). Different letters (a,b) indicate significant differences (*p* < 0.05). SFBS5: DMEM + 10 µg/mL insulin + 5% FBS; SBLH25: SFBS5 + 25 µg/mL BLH; SBLH50: SFBS5 + 50 µg/mL BLH; SBLH100: SFBS5 + 100 µg/mL BLH; SBLH200: SFBS5 + 200 µg/mL BLH; SBLH300: SFBS5 + 300 µg/mL BLH.

Figure 9A,B illustrate images of SVFs differentiated for 1 and 8 days in the presence of varying concentrations of BLH. The morphology of differentiated SVF cells suggested that adipocyte formation occurred stably, as indicated by the presence of lipid droplets. To determine the effect of BLH on the differentiation of chicken SVFs, we measured the expression levels of genes related to SVF differentiation (Figure 9C). Relative mRNA levels of *CEBPB*, a marker for adipogenic differentiation, were the highest in the SFBS5 group (*p* < 0.05), with all BLH-treated groups showing equal or lower levels compared to the control group. In the early stages of adipocyte differentiation, extracellular signal-regulated kinase (ERK) and phosphatidylinositide 3-kinase (PI3K)/Akt regulate the CCAAT/enhancer binding proteins β (C/EBPβ). Generally, in the committed pre-adipocyte stage of in vitro adipogenesis, the *CEBPB* gene is upregulated [52]. Since the *CEBPB* gene plays a crucial role in the early stages of lipid droplet formation, it is presumed that BLH inhibits the early differentiation of SVFs.

**Figure 10:**
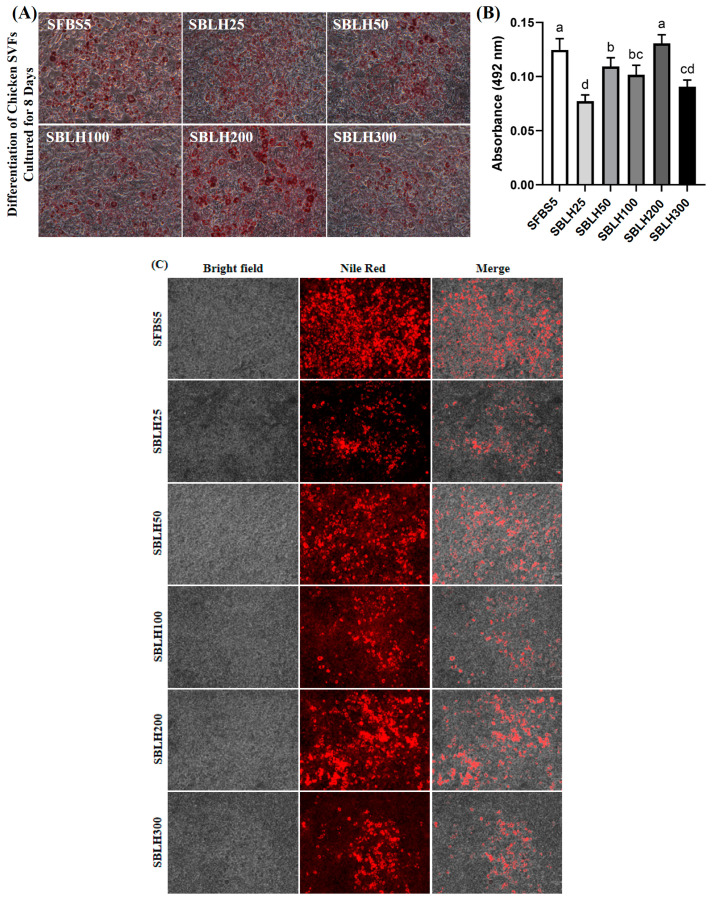
Lipid accumulation analysis of differentiation chicken stromal vascular fractions. (**A**) Oil Red O staining images (magnification: ×200) and (**B**) quantification of differentiation staining in a stromal vascular fraction (SVF) under differentiation conditions for 8 days with BLH treatment. (**C**) Nile Red staining images (magnification: ×10) of stromal vascular fractions in a differentiation culture for 8 days with the addition of BLH. Data are presented as mean ± SD from three biological replicates (n = 3). Different letters (a–d) indicate significant differences (*p* < 0.05). SFBS5: DMEM + 10 µg/mL insulin + 5% FBS; SBLH25: SFBS5 + 25 µg/mL BLH; SBLH50: SFBS5 + 50 µg/mL BLH; SBLH100: SFBS5 + 100 µg/mL BLH; SBLH200: SFBS5 + 200 µg/mL BLH; SBLH300: SFBS5 + 300 µg/mL BLH.

Differentiation induction was performed to verify the adipogenic characteristics of primary cells isolated from abdominal fat tissue and confirm their differentiation into adipocytes. The differentiation of SVF cells into adipocytes was verified by identifying lipid droplets using Oil Red O staining and Nile Red staining (Figure 10A,C). The differentiation degree of SVFs cultured for 8 days was assessed using Oil Red O staining (Figure 10A,B). Only the BLH200 group showed a level of differentiation similar to the SFBS5, while all other treatment groups exhibited significantly lower values than the SFBS5 (*p* < 0.05). Figure 10C illustrates the differentiation capacity (assessed through Nile Red staining) of SVFs supplemented with BLH and cultured for 8 days. The SFBS5 group exhibited a higher differentiation capacity, consistent with results observed in Figure 10A, using Oil Red O staining. The results shown in Figure 10 suggest that BLH can inhibit the lipid droplet formation of chicken SVFs. In the later stages of adipocyte differentiation, transcription factors such as CCAAT/enhancer-binding protein α (C/EBPα) and peroxisome proliferator-activated receptor γ (PPARγ) promote adipocyte maturation. Among these, PPARγ, C/EBPα, and C/EBPβ, which play pivotal roles in adipogenesis, activate the expression of numerous genes involved in lipid accumulation and insulin sensitivity. These transcription factors are upregulated during adipocyte differentiation and are associated with the expression of adipogenic proteins [53]. Based on the results of Figure 9 and Figure 10, this suggests that the suppression of differentiation-related gene expression may have influenced the formation of mature adipocytes and the accumulation of lipid droplets. In the results of Figure 9C and Figure 10B, the BLH-treated groups either had no effect on SVF differentiation or tended to inhibit differentiation compared to the SFBS5 group, with differences observed among the BLH-treatment groups. Figure 9C measures *CEBPB*, a gene expressed in the early stages of SVF differentiation, whereas Figure 10B quantifies lipid droplets, which may account for the slight differences among the treatment groups. A previous study reported that black soldier fly larvae extract inhibited the differentiation of 3T3-L1 pre-adipocytes, suggesting its potential as an anti-obesity functional material, consistent with the findings of [33]. Although there are differences in the extraction methods, the results shown in Figure 10 are consistent with the previous literature, demonstrating that BLH also inhibits the differentiation of SVFs.

## 4. Conclusions

This study demonstrated the potential for BLH to enhance cell growth in chicken primary cell cultures. The addition of BLH up to a concentration of 300 µg/mL was found to be non-toxic to in vitro cultures of chicken leg and breast muscle cells. BLH at a concentration of 100 µg/mL provided significant benefits for the proliferation of chicken primary muscle cells. Additionally, in chicken myogenic cells, BLH enhances proliferation at low concentrations during short-term culture (e.g., 3 days) and at high concentrations during long-term culture (e.g., 7 days) (Figure 5, Figure 6 and Figure 7). The proliferative effect was more pronounced when BLH was added at high concentrations. This effect is presumed to be due to the ability of glutamic acid in BLH to mitigate ammonia and lactate accumulation in the culture medium [47,49]. Managing waste metabolites is a crucial aspect of the cultured meat industry [52,54]. This could serve as a key advantage in addressing ammonia and lactate accumulation, which is a major challenge in large-scale production and long-term culture, both essential processes in cultured meat production [55]. BLH has great potential in terms of enhancing the efficiency of culture media utilization. Similarly, the proliferation of chicken SVFs was promoted with BLH concentrations of up to 300 µg/mL without exhibiting toxicity (Figure 8). These findings indicate that BLH has the potential to be used as a proliferation-promoting supplement for cultivated meat production using primary chicken cells. However, BLH was found to inhibit the differentiation of SVF cells (Figure 9 and Figure 10). Further comprehensive investigations and optimization are essential for the sustained expansion of cells. Additionally, for the production of cultured meat, various types of cells, such as muscle cells and fat cells, need to proliferate, differentiate, and mature at different stages of growth, each fulfilling their specific role [56,57]. The addition of hydrolysates appears to be effective at low concentrations, while high concentrations have been reported to cause growth inhibition [12]. BLH supplementation was observed to inhibit the differentiation of chicken SVFs. Since BLH does not promote the growth of all primary cells at every stage, it requires selective and appropriate application. Future research should focus on elucidating the precise molecular mechanisms underlying BLH’s effects on muscle and fat cells, as well as its scalability for industrial applications. By addressing these aspects, BLH could contribute significantly to the development of sustainable cultured meat and edible insect industries. In conclusion, BLH can be utilized as a supplement in culture media to enhance the proliferation of primary cells isolated from broilers. The use of BLH demonstrated the potential for application in cultured meat production, highlighting its role in promoting the proliferation of primary cells isolated from broilers. This suggests that BLH could contribute to the development of both the edible insect and cultured meat industries. Further research is needed to expand and optimize the potential applications of BLH, which could lead to various industrial uses, such as mitigating the accumulation of metabolic byproducts in culture systems.

## Figures and Tables

**Table 1:** Sequences of primers used in the RT-qPCR.

Primer	Description	Direction	Sequence (5′-3′)
*PDGFRA*	Platelet-derived growth factor receptor alpha	F	GAGCTTGGCAAAAGGAACAG
		R	GATCCGAGGAGTCAATTCCA
*ITGB1*	Integrin subunit beta 1	F	AGTGGTATGATGCCAAGGAA
		R	GTTTCCATCCTCTCCCATCT
*MYOD1*	Myogenic differentiation 1	F	TATTACCCCTGTTCTGGCCA
		R	GCACAACAAACCAAGCAACA
*PAX7*	Paired box 7	F	CAGGTGGAACCTCACCATAG
		R	AGGTGGGAGGACAGTAGGAC
*NCAM1*	Neural cell adhesion molecule 1	F	TTCCATCACGTGGAAAACTT
		R	CTTGGGAGCATACTGCACTT
*MYF5*	Myogenic factor 5	F	AGATGGAGGTGATGGACAGC
		R	GGACGTGTTCCTCTTCCTCA
*CEBPB*	CCAAT enhancer binding protein beta	F	GACAAGCACAGCGACGAGTA
		R	CACCTTCTTCTGCAGCCTCT
*ACTB*	Actin beta	F	AATGGCTCCGGTATGTGCAA
		R	GGCCCATACCAACCATCACA

## Data Availability

The original contributions presented in the study are included in the article, further inquiries can be directed to the corresponding author.
